# The knowledge, awareness and practices of radiation safety amongst orthopaedic surgeons

**DOI:** 10.4102/sajr.v24i1.1806

**Published:** 2020-02-27

**Authors:** Louis W.A. van Papendorp, Farhana E. Suleman, Heleen Hanekom

**Affiliations:** 1 Department of Radiology, Faculty of Health Sciences, University of Pretoria and Steve Biko Academic Hospital, Pretoria, South Africa; 2 Department of Radiology, Faculty of Health Sciences, University of Pretoria and Kalafong Hospital, Pretoria, South Africa

**Keywords:** radiation protection, orthopaedic surgeon, radiation safety, knowledge, awareness, practices

## Abstract

**Background:**

Fluoroscopic imaging in orthopaedic theatres is increasing, with added risk to the orthopaedic surgeon who is increasingly being exposed to ionising radiation. It is thus crucial for orthopaedic surgeons to have a working knowledge of radiation safety. In spite of these concerns, however, many orthopaedic surgeons do not receive standard training in radiation safety.

**Objectives:**

The evaluation of orthopaedic surgeons’ knowledge, awareness and everyday practices regarding radiation safety in an academic hospital.

**Methods:**

A questionnaire with multiple-choice-type questions was developed by a panel of experts and used to conduct a descriptive study. The questionnaire had multiple dimensions, each evaluating orthopaedic knowledge, awareness and practices, respectively. The study population included orthopaedic surgeons rotating within the orthopaedic circuit of the University of Pretoria.

**Results:**

Orthopaedic surgeons regularly make use of fluoroscopic imaging in theatre, with 34 (77%) participants indicating that they use fluoroscopy in more than half of all their procedures performed. Most participants have insufficient knowledge of radiation safety, with the majority failing to correctly answer basic questions on radiation safety. Forty (91%) participants do not wear personal dosimeters, in spite of 39 participants (89%) believing that they are vulnerable to adverse effects. Basic radiation protection devices are underutilised, with 32 (73%) participants indicating that they have not received adequate training in radiation safety.

**Conclusion:**

The majority of orthopaedic surgeons regularly use fluoroscopic imaging in theatre yet lack in-depth knowledge and awareness regarding radiation safety associated with this imaging modality. Implementation of a radiation safety training programme is thus recommended.

## Introduction

The use of fluoroscopic imaging is increasing in the modern orthopaedic theatre.^1^ Benefits of intra-operative fluoroscopy include the indirect visualisation of anatomy, enabling many orthopaedic procedures to be performed with greater ease, in less time and with less traumatisation of patient tissues, thus reducing patient morbidity.^2,3,4^

Results from a survey analysis conducted by Tunçer et al.^4^ confirmed that the need for fluoroscopy was indeed very high in the orthopaedic theatre. Placement of internal and external fixation devices, as well as long bone fracture reductions, are amongst the orthopaedic procedures frequently performed under fluoroscopic guidance.^1^

Fluoroscopic use in the theatre setting is, however, not without risk to the orthopaedic surgeon, the biological effects of ionising radiation being well-known.^3,5^ These effects include dose-dependent deterministic effects and dose-independent stochastic effects.^5,6^ Deterministic effects are unlikely to occur below a specific dose threshold and include cataracts, alopecia, headache, dermal ulceration and infertility. Stochastic effects do not have a threshold dose and may include the induction of malignancy in radiosensitive organs such as the breasts, lungs, thyroid and red bone marrow.^1^

Troisi et al.^1^ proved that the annual dosimeter readings from a group of orthopaedic registrars in the Pietermaritzburg Complex did not exceed the recommended dose limits, as set out by the International Commission on Radiological Protection. This renders the orthopaedic registrars of the Pietermaritzburg Complex unlikely to develop deterministic effects but does not exclude their risk of developing stochastic effects secondary to chronic exposure to low levels of ionising radiation in the orthopaedic theatre.

The linear no-threshold model states that the long-term risk of cancer induction is directly proportional to the sum of all the dosages acquired in an individual’s lifetime and that all exposures to ionising radiation, no matter how small, should be regarded as harmful.^6^ It is thus crucial for orthopaedic surgeons to have a working knowledge of radiation safety, in order to keep exposure in theatre as low as reasonably possible.

Furthermore, ionising radiation is both invisible and intangible, rendering it a hazard, difficult to stay aware of. It unfortunately remains unavoidable for the orthopaedic surgeon who operates in close proximity to the x-ray beam.^7,8^ In spite of the above-mentioned concerns, many orthopaedic surgeons do not receive standard training in radiation safety.^9^ Similar previous studies by Tunçer et al.,^4^ Saroki et al.^7^ and Nugent et al.^10^ all concluded that orthopaedic surgeons have inadequate knowledge concerning the use and risks of ionising radiation, as well as lack the necessary radioprotective knowledge for preventing damage caused by ionising radiation. Study objectives thus included the assessment of orthopaedic surgeons’ knowledge, awareness and everyday practices regarding radiation safety in an academic hospital.

## Research methods and design

### Study design and population

A descriptive study was conducted. The questionnaire was completed by 44 of the 56 orthopaedic surgeons actively working within the University of Pretoria’s orthopaedic circuit, yielding a response rate of 79%. Participants included five medical officers, nine junior registrars, 20 senior registrars, nine consultants and one professor. The majority of the participants (46%) were senior registrars; participants were considered as senior on completion of at least 2 years of training. Exclusion criteria included female participants who were pregnant at the time of data collection. Five questionnaires were incompletely filled in and were excluded from the study.

### Study setting and sample size

The study was set within the orthopaedic circuit of the University of Pretoria, which included participants from Steve Biko, Kalafong and One Military Hospitals in Pretoria. All participants were included (total of 56 orthopaedic surgeons working within the circuit) and no sampling was done.

### Data collection tools

A panel of experts, including radiologists, orthopaedic surgeons, a biological statistician, and previous literature helped develop our questionnaire, which is available as Online Appendix 1. The questionnaire is non-validated and the correct answers are indicated by an asterisk adjacent to the corresponding option.

The final questionnaire included 14 questions with responses framed in the following ways: multiple choice with single answer, multiple choice with single answer and text entry, multiple choice with multiple answers and multiple choice with multiple answers and text entry. The questionnaire had multiple dimensions – the first part included questions pertaining to participant level of training, the frequency of and the necessity for the use of fluoroscopy in theatre. The second part assessed adequacy of radiation safety training, while the third part evaluated participant knowledge. Questions evaluating radiation safety knowledge tested participants on fundamental radiation principles, some of which could directly be applied in the orthopaedic theatre to reduce radiation dose. These included basic principles such as knowledge of the ‘As Low as Reasonably Achievable’ (ALARA) principle, the inverse square law, annual radiation dose limits for radiation workers and the methods of radiation dose reduction. The last part assessed participant radiation safety awareness, personal dosimeter use and everyday practices regarding radiation protection devices.

Questionnaires were distributed at weekly orthopaedic academic meetings over a period of 1 month – data collection started on 04 September 2018 and was terminated on 02 October 2018. Participants were allowed 1 h to complete the questionnaire.

### Data analysis

A descriptive, univariate analysis was performed, with assessment of one variable at a time. Focus was on the distribution of participants for each individual variable, with data expressed as percentages of the total study population, and displayed graphically.

### Study limitations

Five questionnaires were left partially completed after termination of data collection. This is a limitation of using the hard copy mode of delivery, as participants may decide to leave specific questions unanswered. Incomplete questionnaires were excluded, thus resulting in a smaller study population.

A further limitation was not including a more widespread group of professionals (radiographers, medical physicists and radiation safety officers) in the development of the questionnaire. This would have added more depth in terms of radiation safety expertise and thus question relevance.

### Ethical consideration

Participation in this study was completely voluntary, and questionnaires were completed anonymously. No incentives were offered to participants. The study received ethical clearance from the University of Pretoria’s Health Ethics Committee, with ethics number 211/2018.

## Results

**FIGURE 1:**
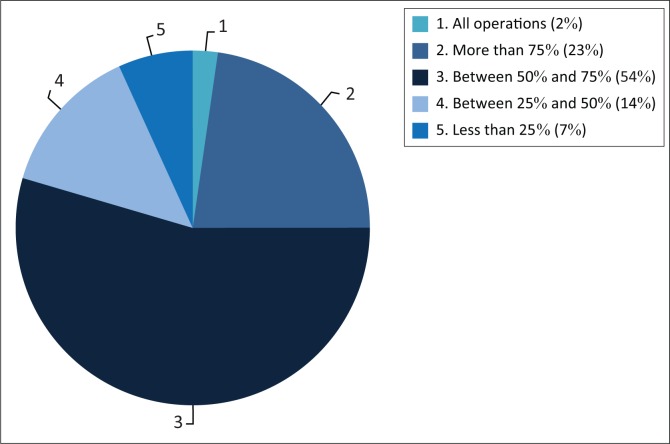
Total amount of fluoroscopic use in theatre (*N* = 44).

Orthopaedic surgeons regularly use fluoroscopic imaging in theatre, with 34 (77%) participants indicating that they use fluoroscopy in over 50% of their daily operations. All participants indicated that fluoroscopic imaging is necessary to more effectively execute certain orthopaedic procedures (Figure 1).

Of the surveyed orthopaedic surgeons, 32 (73%) felt that they had not received adequate training on radiation safety. A question evaluating which prior sources of radiation safety training the participants had accessed allowed multiple options to be marked. Options included a radiation protection course, lecture by someone with training in radiation safety, discussion with colleagues, undergraduate training, Internet, none and other. ‘Discussion with colleagues’ was the source most indicated to obtain knowledge on radiation safety, accounting for 15 (34%) of the participants. Ten (23%) participants indicated that they had not accessed any sources on radiation safety whatsoever.

Questions evaluating radiation safety knowledge revealed that 34 (77%) participants did not know the meaning of the ALARA principle. Thirty-five (80%) participants were unable to correctly identify the annual dose limit for classified radiation workers, in spite of 40 (91%) participants indicating that orthopaedic surgeons should be classified as radiation workers. Thirty-one (70%) participants were able to identify the correct definition of the inverse square law.

**FIGURE 2:**
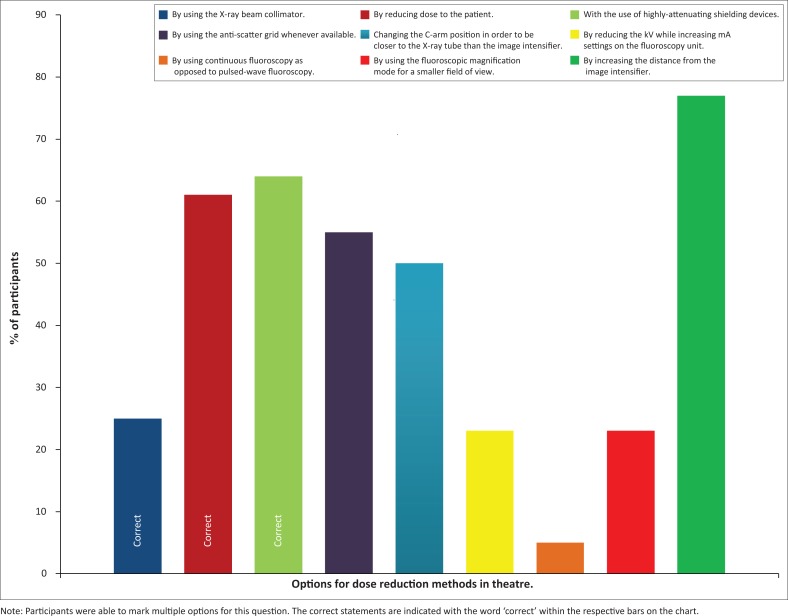
Different options of dose reduction in the orthopaedic theatre.

Another question asked participants to identify basic methods of dose reduction in the orthopaedic theatre. Nine different options were provided at random, and participants were urged to mark all the relevant options. The different question options along with the percentage of participants who indicated each option are demonstrated in Figure 2.

Seventeen (39%) participants did not know that lowering the dose to the patient would lower the dose to the fluoroscopy operator, while 16 (36%) participants did not know that the use of highly attenuating shielding devices would similarly lower the dose. The focus should, however, not be on the above participants who failed to identify the correct methods of dose reduction but rather on the participants who indicated incorrect methods that they thought would reduce dose, rendering more harm. Twenty-four (55%) participants thought that the use of an anti-scatter grid would reduce dose, while 22 (50%) of them thought that standing closer to the x-ray tube itself will be more protective. There were no participants who correctly answered all of the questions on radiation safety knowledge.

The section on radiation awareness revealed that 39 (89%) participants believed that they were at risk of developing adverse effects secondary to fluoroscopic radiation exposure. This while only two (5%) participants always checked the amount of screening time after completing their procedures.

**FIGURE 3:**
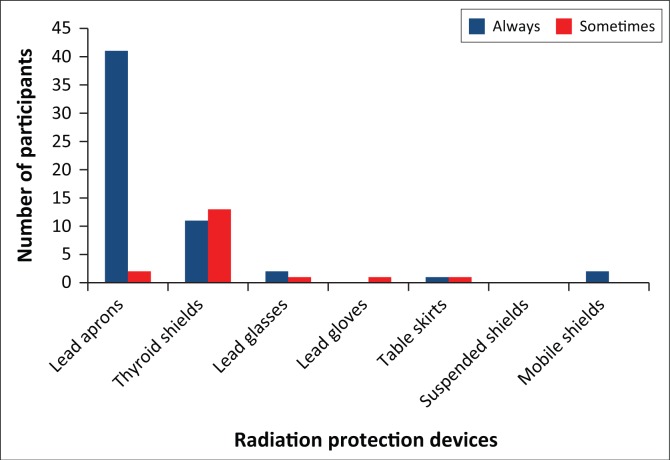
Orthopaedic use of radiation protection devices.

Pertaining to the everyday practices of the study participants, only three (7%) made use of personal dosimeters. Most participants (93%) always made use of lead aprons and 11 (25%) participants always made use of thyroid shields when screening in theatre. Two (5%) participants only sometimes made use of lead aprons and 13 (30%) participants only sometimes made use of thyroid shields, as depicted in Figure 3. This while lead aprons and thyroid shields are the most basic forms of radiation protection devices and are considered indispensable in primary radiation protection.

**FIGURE 4:**
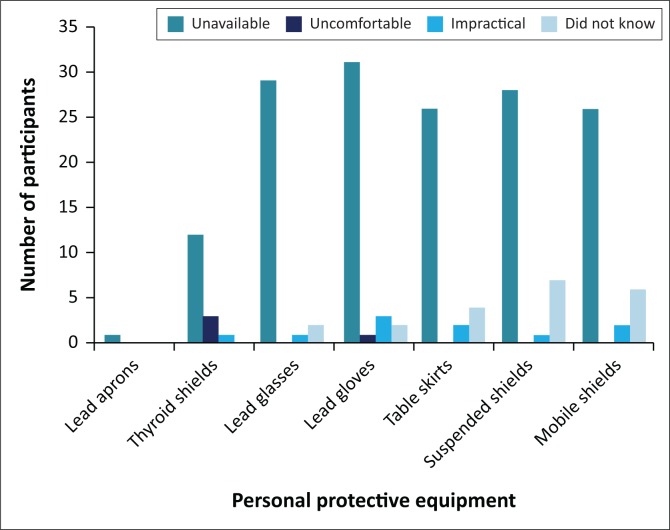
Reasons for not using radiation protection devices.

Personal protective devices like lead glasses, lead gloves, lead table skirts, and fixed and mobile lead shields were mostly deemed unavailable. Other reasons indicated for not using certain radiation protection devices included discomfort and impracticality, as demonstrated in Figure 4. Included in the figure are the number of orthopaedic participants who were unaware that a particular device was meant for radiation protection.

## Discussion

The use of fluoroscopic imaging in the orthopaedic theatre is necessary but not without the risk of exposure to ionising radiation. In order to minimise the risk to the fluoroscopic operator and the theatre staff, adequate knowledge and awareness pertaining to radiation safety are required. Based on this study’s findings, radiation safety knowledge is clearly insufficient amongst orthopaedic surgical staff.

Gendelberg et al.^11^ showed that after attending a structured radiation safety programme, orthopaedic registrars were able to reduce radiation time and exposure while operating, resulting in decreased radiation exposure to registrars and patients. Similarly, the implementation of such a radiation safety training programme might benefit the participants of this study.

The results of this study reveal a lack of radiation safety awareness amongst the participants, with radiation-shielding devices being underutilised, the majority of participants not monitoring their amount of screening time and very few actually wearing personal dosimeters when screening. This complacent attitude amongst participants may have originated from a sense of safety as proclaimed by certain previous studies. A related study performed in the Pietermaritzburg training circuit by Troisi et al.^1^ measured the exposure on orthopaedic surgeons’ personal dosimeters and concluded that orthopaedic dosages were still within international safety limits. Results like these are, however, not a justification to be less aware about radiation safety.

With many radiation protection devices deemed unavailable, it leaves the following question open for further discussion: ‘Would the orthopaedic surgeons have used these devices if they were readily available?’ Furthermore, participants indicating that they were unaware that certain devices were an option for radiation protection, substantiates both the participants’ lack of radiation knowledge and awareness.

A study conducted by Meisinger et al.^12^ revealed possible reasons for operators not making use of certain radiation protection devices. Amongst others, awkward positioning of shields, heavy weight of garments, tight-fitting thyroid collars and rigid lead gloves complement the findings of impracticality and discomfort demonstrated in this study.

Similar studies conducted in the United States, Ireland and Turkey confirmed analogous results, concluding that the need for fluoroscopy was very high in the orthopaedic theatre but that orthopaedic surgeons have inadequate knowledge about the risks of fluoroscopy and the methods for preventing biological damage.^4,7,10^

## Conclusion

The majority of orthopaedic surgeons regularly use fluoroscopic imaging in theatre, yet lack in-depth knowledge and awareness regarding radiation safety associated with this imaging modality. This while personal protective equipment are either unavailable or underutilised when present.

It can thus be recommended that a radiation safety and protection training programme be implemented within the orthopaedic circuit of the University of Pretoria.
